# Supplementary material for: A Diagnostic Model to Predict SARS-CoV-2 Positivity in Emergency Department Using Routine Admission Hematological Parameters
Source: Diagnostics (Basel). 2021 Aug 28;11(9):1566. doi: 10.3390/diagnostics11091566 (PMC8471783; doi:10.3390/diagnostics11091566)
Supplement: Supplementary file 1 [file diagnostics-11-01566-s001.zip › diagnostics-1301903-supplementary.pdf]

diagnostics

**SUPPLEMENTARY MATERIAL**

**Figure S1. Patients' flow-chart.**

Diagnosis was suspected but not proven when swabs results were doubtful and was not possible to obtain a certain diagnosis in ED, or when patients died at admission before performing COVID-19 swab. Even if these patients showed typical signs and symptoms or chest imaging suggestive for COVID-19 disease, they were excluded from further analysis due to uncertain diagnosis. For negative controls, mismatch between swab results and serum immunoglobulin tests was intended as negative swab with SARS-COV-2 IgM and/or IgG found positive. These patients were unlikely to be considered totally COVID-19 free and were excluded.

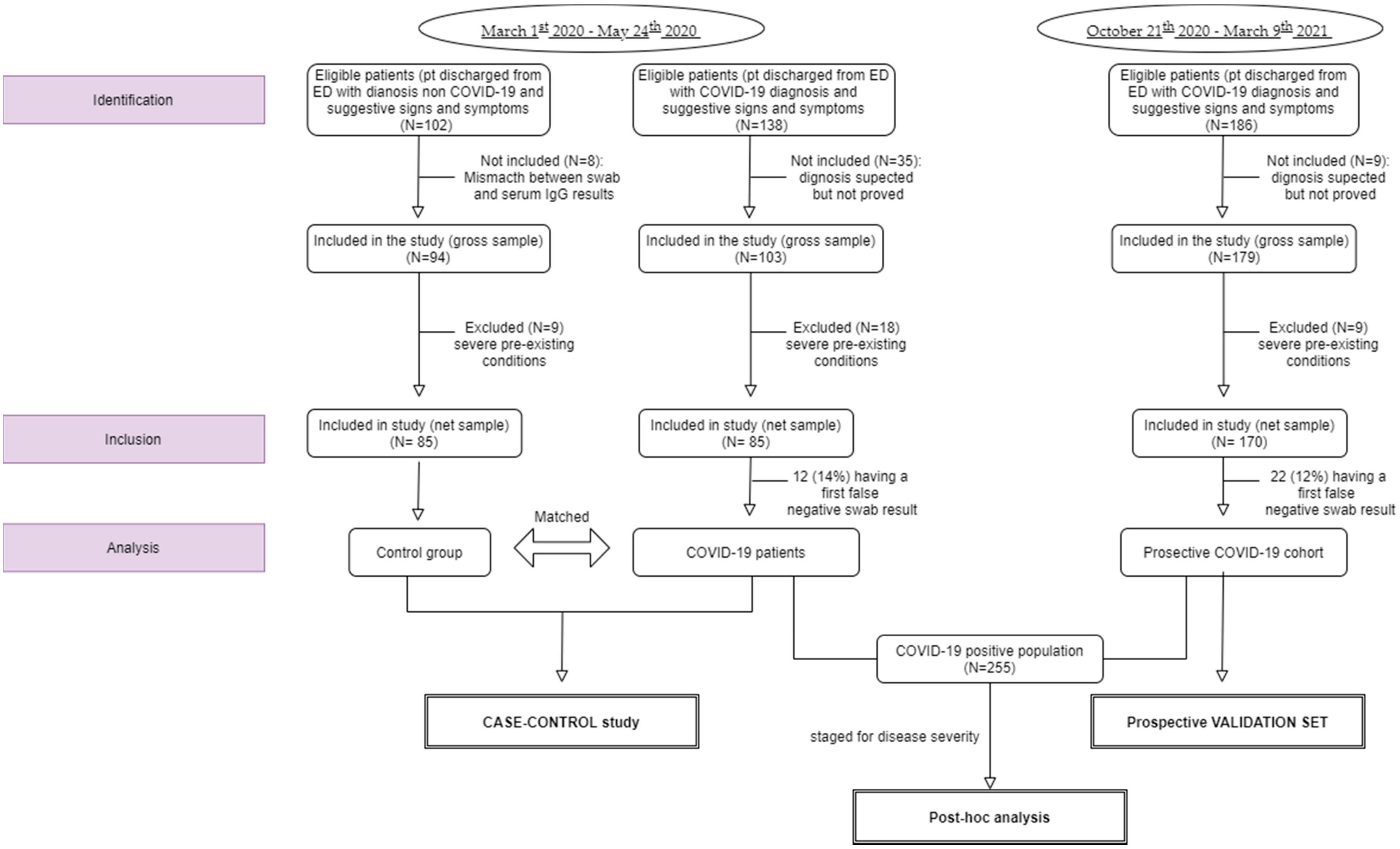

**Table S1. Etiology of admission in ED of control group.** COPD, chronic obstructive pulmonary disease; URTI, Upper respiratory tract infections; ORL, otorinolaryngoiatric; CNS, central nervous system; EBM, Epstein-Barr virus; CMV, cytomegalovirus; UTI, urinary tract infection.

| Symptoms of admission (% on 85 controls) | Etiology | Frequency (n) | Percent (%) |
|---|---|---|---|
| Dyspnea (40%) | Pneumotorax | 2 | 5,9 |
| | Non cardiogenic pulmonary edema | 3 | 8,8 |
| | Pain attack | 1 | 2,9 |
| | Acute heart failure | 4 | 11,8 |
| | Bacterial pnemonia | 11 | 32,4 |
| | Pulmonary embolism | 2 | 5,9 |
| | COPD exacerbation | 4 | 11,8 |
| | Other viral pneumonia | 4 | 11,8 |
| | Pulmonary autoimmune disorders | 2 | 5,9 |
| | Anaphylaxis | 1 | 2,9 |
| | Total | 34 | 100,0 |
| Fever (34.1%) | URTI and ORL infections | 4 | 13,8 |
| | Colds | 3 | 10,3 |
| | Lung bacterial, viral of fungal infections | 2 | 6,9 |
| | Heart infections (pericarditis, endocarditis, myocarditis) | 4 | 13,8 |
| | CNS infections | 1 | 3,4 |
| | Systemic viral infections (EBV, CMV, flu) | 6 | 20,7 |
| | Sepsis of unknown origin | 1 | 3,4 |
| | Rheumatological disorders | 2 | 6,9 |
| | Haematological disorders | 1 | 3,4 |
| | UTI | 4 | 13,8 |
| | Soft tissues infections | 1 | 3,4 |
| | Total | 29 | 100,0 |
| Gastroenteric symptoms (21.2%) | Viral gastroenteritis | 3 | 16,7 |
| | Alimentary toxinfections | 2 | 11,1 |
| | Acute abdominal infections | 8 | 44,4 |
| | Inflammatory bowel disease | 1 | 5,6 |
| | Cancer | 1 | 5,6 |
| | Renal infection | 3 | 16,7 |
| | Total | 18 | 100,0 |
| Aspecific flu-like symptoms with Covid-like pattern at chest CT-scan (4.7%) | Atypical Pneumonia | 2 | 50 |
| | Alveolitis | 2 | 50 |
| | Total | 4 | 100,00 |

**Table S2. Univariate analysis of all hematological parameters performed.** AST, aspartate aminotransferase; ALT, alanine aminotransferase; GGT, gamma glutamyl transpeptidase; CPK, creatine phosphokinase ; HSCT, high-sensitivity cardiac troponin T; PT sec, prothrombin time (seconds); PT%, prothrombin time percentage; INR, international normalized ratio; PTT sec, partial prothrombin time (seconds); PTT%, partial prothrombin time in percentage; WLR, absolute count of white blood cells to absolute count of lymphocytes ratio; NLR, absolute count of neutrophils to absolute count of lymphocytes ratio; DELR, d-dimer to the sum of lymphocytes percentage and eosinophils percentage ratio; WBC, white blood cell count; RBC, red blood cell count; MCV, mean corpuscular volume; MCH, mean corpuscular hemoglobin; MCHC, mean corpuscular hemoglobin concentration; RDW, red cell distribution width; LUC, large unstained cells; HDW, hemoglobin distribution width.

| Analyte | Controls group | | | COVID-19 patients | | | P-value |
|---|---|---|---|---|---|---|---|
| | N | Mean | SD | N | Mean | SD | |
| **Proteins (g/dl)** | 79 | 7.26 | 0.57 | 77 | 7.11 | 0.87 | > 0.05 |
| **Albumin (g/dl)** | 70 | 3.71 | 0.45 | 74 | 3.1 | 0.58 | **< 0.001** |
| **Glycemia (mg/dl)** | 85 | 108 | 30 | 79 | 119 | 34 | **< 0.001** |
| **Blood Urea Nitrogen (mg/dl)** | 85 | 18.45 | 10.62 | 79 | 28.70 | 24.92 | **0.007** |
| **Creatinin (mg/dl)** | 85 | 0.87 | 0.29 | 76 | 1.08 | 0.51 | **0.004*** |
| **Uric acid (mg/dl)** | 46 | 5.25 | 1.67 | 39 | 5.11 | 2.26 | < 0.05 |
| **Total bilirubin (mg/dl)** | 84 | 0.62 | 0.34 | 78 | 0.64 | 0.4 | > 0.05 |
| **Direct bilirubin (mg/dl)** | 83 | 0.18 | 0.16 | 78 | 0.21 | 0.16 | **0.039*** |
| **AST (U/l)** | 84 | 26.86 | 16.33 | 78 | 41.09 | 30.89 | **< 0.001*** |
| **ALT (U/l)** | 84 | 33.68 | 22.77 | 78 | 38.63 | 34.69 | > 0.05 |
| **Alkaline phosphatase (U/l)** | 63 | 76.38 | 25.18 | 61 | 77.23 | 28.98 | > 0.05 |
| **Lactic dehydrogenasis (U/l)** | 79 | 209.46 | 72.08 | 84 | 329.82 | 136.45 | **< 0.001*** |
| **GGT (U/l)** | 75 | 37.59 | 38.47 | 73 | 46.97 | 52.58 | > 0.05 |
| **CPK (U/l)** | 72 | 116.46 | 84.15 | 72 | 129.79 | 103.58 | **0.036** |
| **Cholinesterase (U/l)** | 59 | 11244 | 3061 | 58 | 10239 | 3725 | > 0.05 |
| **Amylase (U/l)** | 81 | 58.07 | 21.87 | 80 | 58.69 | 29.65 | > 0.05 |
| **Lipase (U/l)** | 82 | 137.80 | 92.93 | 74 | 163.26 | 91.66 | **0.022*** |
| **C reactive protein (mg/dl)** | 81 | 0.67 | 0.87 | 80 | 6.52 | 5.97 | **< 0.001*** |
| **Calcium (mg/dl)** | 74 | 8.89 | 0.45 | 73 | 8.49 | 0.62 | **< 0.001*** |
| **Phosphorus (mg/dl)** | 52 | 3.56 | 1.04 | 55 | 3.08 | 0.75 | **0.004*** |
| **Magnesium (mg/dl)** | 43 | 2.10 | 0.31 | 51 | 2.14 | 0.39 | > 0.05 |
| **Sodium (mEq/L)** | 84 | 138.70 | 3.19 | 79 | 137.11 | 4.2 | **0.001** |
| **Potassium (mEq/l)** | 84 | 4.12 | 0.53 | 79 | 3.86 | 0.63 | **0.001*** |
| **Chloride (mEq/l)** | 84 | 105.08 | 4.56 | 80 | 101.99 | 6.30 | **< 0.001*** |
| **HSCT (ng/ml)** | 54 | 0.02 | 0.02 | 47 | 0.78 | 3.48 | **0.003** |
| **PT sec** | 79 | 12.17 | 1.18 | 77 | 13.87 | 3.97 | **< 0.001*** |
| **PT %** | 79 | 90.01 | 11.82 | 73 | 75.91 | 14.96 | **< 0.001*** |

| | | | | | | | |
|---|---|---|---|---|---|---|---|
| **INR** | 79 | 1.09 | 0.11 | 76 | 1.19 | 0.17 | **< 0.001*** |
| **PTT sec** | 79 | 32.55 | 4.02 | 78 | 34.68 | 7.86 | > 0.05 |
| **PTT %** | 79 | 1.05 | 0.13 | 76 | 1.09 | 0.21 | > 0.05 |
| **Fibrinogen (mg%)** | 66 | 329.39 | 119.62 | 75 | 569.48 | 197.41 | **< 0.001*** |
| **D-dimers (ng/ml)** | 36 | 125.14 | 91.56 | 49 | 1924.67 | 3477.69 | **< 0.001*** |
| **Procalcitonin (ng/ml)** | 18 | 0.10 | 0.23 | 72 | 0.3 | 0.49 | **< 0.001*** |
| **WLR** | 84 | 4.09 | 2.12 | 73 | 11.87 | 11.67 | **< 0.001** |
| **NLR** | 84 | 2.69 | 2.02 | 73 | 9.44 | 9.99 | **<0.001** |
| **DLER** | 35 | 4.21 | 3.52 | 46 | 286.72 | 646.47 | **< 0.001** |
| **WBC ($X10^3/\mu L$)** | 84 | 7.65 | 2.27 | 76 | 7.75 | 3.8 | **0.003** |
| **RBC ($x10^6/\mu L$)** | 84 | 4.69 | 0.57 | 79 | 4.6 | 0.76 | > 0.05 |
| **Hemoglobin (g/dl)** | 84 | 13.71 | 1.92 | 78 | 13.33 | 2.01 | > 0.05 |
| **Hematocrit (%)** | 84 | 41.26 | 5.16 | 79 | 39.22 | 5.93 | **0.031*** |
| **MCV (fL)** | 84 | 88.17 | 7.04 | 78 | 86.43 | 4.92 | **0.005*** |
| **MCH (pg/cell)** | 84 | 29.88 | 6.8 | 80 | 29.05 | 2.35 | **0.032** |
| **MCHC (g/dL)** | 84 | 33.11 | 1.40 | 80 | 33.65 | 1.21 | **0.010*** |
| **RDW (%)** | 84 | 14.05 | 1.48 | 79 | 14.46 | 1.76 | > 0.05 |
| **Platelets ($x10^3/\mu L$)** | 84 | 236.76 | 69.26 | 81 | 201.30 | 75.1 | **0.002** |
| **Neutrophils (%)** | 84 | 61.80 | 9.76 | 80 | 74.6 | 14.10 | **< 0.001*** |
| **Lymphocytes (%)** | 83 | 27.97 | 8.31 | 79 | 14.90 | 9.53 | **< 0.001*** |
| **Monocytes (%)** | 82 | 5.81 | 1.49 | 77 | 5.32 | 2.54 | **0.003** |
| **Eosinophils (%)** | 84 | 2.36 | 1.73 | 79 | 0.41 | 0.44 | **< 0.001*** |
| **Basophils (%)** | 84 | 0.47 | 0.22 | 79 | 0.45 | 0.38 | **0.009** |
| **LUC (%)** | 84 | 1.69 | 0.61 | 80 | 1.4 | 0.74 | **< 0.001*** |
| **Neutrophils Abs Count ($x10^3/\mu L$)** | 84 | 4.79 | 1.75 | 76 | 6.24 | 3.88 | **0.018** |
| **Lymphocytes Abs Count ($x10^3/\mu L$)** | 84 | 2.09 | 0.85 | 78 | .87 | 0.38 | **< 0.001*** |
| **Monocytes Abs Count ($x10^3/\mu L$)** | 84 | 0.45 | 0.18 | 79 | 0.44 | 0.31 | **0.023** |
| **Eosinophils Abs Count ($x10^3/\mu L$)** | 84 | 0.18 | 0.14 | 81 | 0.05 | 0.09 | **< 0.001*** |
| **Basophils Abs Count ($x10^3/\mu L$)** | 84 | 0.04 | 0.04 | 80 | 0.03 | 0.04 | > 0.05 |
| **LUC Abs Count ($x10^3/\mu L$)** | 84 | 0.12 | 0.05 | 80 | 0.1 | 0.05 | **< 0.001** |
| **HDW (g/dl)** | 84 | 2.69 | 0.38 | 79 | 2.91 | 0.37 | **< 0.001*** |

*two-sided P-value. Alfa significance < 0.05

**Figure S2. ROC curves for eosinophil percentage and absolute count, NLR, DELR, and WLR.**
**a.** ROC curve for eosinophil percentage and for eosinophil absolute count. **b** ROC curve for NLR. **c** ROC curve for DELR and for WLR.

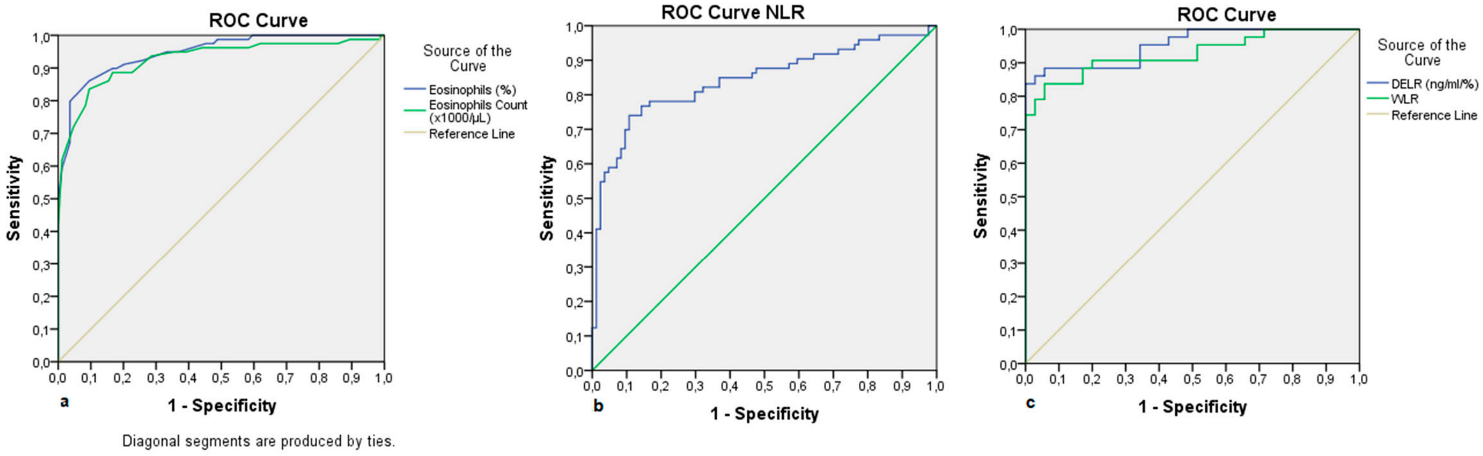


**Figure S2.** ROC curve analysis for eosinophils percentage and absolute count, NLR, DELR, WLR.

**Table S3. Kendall's tau B non-parametric bivariate correlation.** Correlation coefficients between the analysed haematological 2yparameters and COVID-19, where a coefficient = 1 indicates a perfect positive correlation. All values examined were statistically significant at a 2-tailed P-value < 0.001. The cut-offs used are standard laboratory cut-offs where available and thresholds found through ROC curve analysis for eosinophil percentage and absolute count, NLR, WLR, and DELR.

| Parameter | N | Kendall's tau B Correlation coefficient | Sig. (two-tailed) |
|---|---|---|---|
| **COVID 19** | 170 | 1 | |
| **Hypoalbuminemia** | 145 | 0.519 | <0.0001 |
| **Hyperglycemia** | 164 | 0.279 | <0.0001 |
| **Increased BUN** | 164 | 0.230 | 0.003 |
| **Increased LDH** | 163 | 0.452 | <0.0001 |
| **Increased CRP** | 161 | 0.665 | <0.0001 |
| **Decreased PT percentage** | 152 | 0.347 | <0.0001 |
| **Increased fibrinogen** | 142 | 0.561 | <0.0001 |
| **Increased d-dimers** | 85 | 0.608 | <0.0001 |
| **Increased neutrophils percentage** | 164 | 0.536 | <0.0001 |
| **Decreased lymphocyte percentage** | 162 | 0.635 | <0.0001 |
| **Decreased eosinophil percentage** | 163 | 0.767 | <0.0001 |
| **Decreased lymphocyte count** | 162 | 0.588 | <0.0001 |
| **Decreased eosinophil count** | 165 | 0.723 | <0.0001 |
| **Increased NLR** | 157 | 0.644 | <0.0001 |
| **Increased WLR** | 157 | 0.655 | <0.0001 |
| **Increased DELR** | 81 | 0.841 | <0.0001 |

**Figure S3. ROC curve for Cumulative score in case-control study.** ROC curve for CS: AUC of 0.966; SE 0.005; asymptotic P < 0.001; 95%CI: 0.986-1.000; optimal threshold 6.5 (Youden's index 0.94).

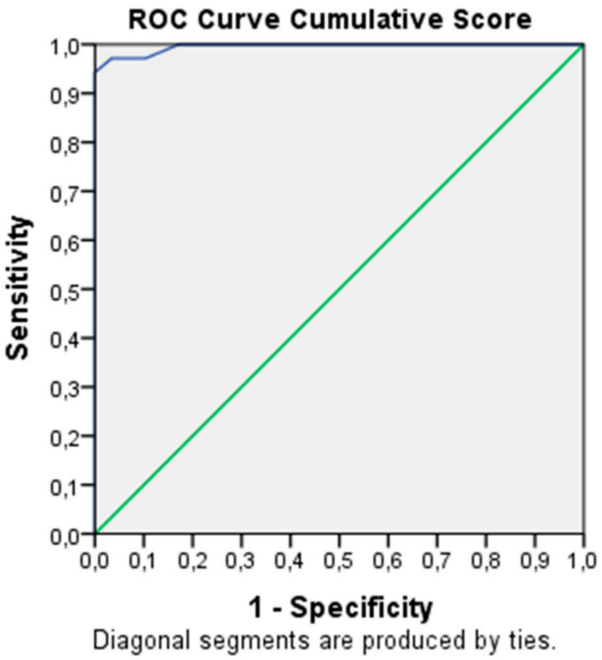


**Figure S3. ROC curve for CS in case-control study**
